# Supplementary material for: Inferring entropy production rate from partially observed Langevin dynamics under coarse-graining
Source: Phys Chem Chem Phys. Author manuscript; Available in PMC 2022 Oct 13. (PMC7613705; doi:10.1039/d2cp03064k)
Supplement: Supplementary Information [file EMS155150-supplement-Supplementary_Information.pdf]

This journal is © the Owner Societies 2022

Supplementary Information

Inferring Entropy Production Rate from partially observed Langevin dynamics under Coarse-Graining

Aishani Ghosal[a] and Gili Bisker[*,a,b,c,d]

[a] Department of Biomedical Engineering, Tel Aviv University, Tel Aviv 6997801, Israel

[b] Center for Physics and Chemistry of Living Systems, Tel-Aviv University, Tel Aviv 6997801, Israel

[c] Center for Nanoscience and Nanotechnology, Tel-Aviv University, Tel Aviv 6997801, Israel

[d] Center for Light-Matter Interaction, Tel-Aviv University, Tel Aviv 6997801, Israel

*Corresponding author: Gili Bisker
Email: bisker@tauex.tau.ac.il

Supplementary Information Text

Simulated trajectories. We simulated Eq. 1 and Eq. 2 for the trajectories of the tip position of the hair bundle (X_1) – the observed variable. The trajectories for different values of the driving parameters (F_{max}, S, and keeping $T_{max} = 1.5\,T$) are plotted in *Figure S1*. These trajectories are later used to calculate the EPR bounds $(\mathrm{EPR_{WTD}})$ on the total EPR $(\mathrm{EPR_{tot}})$ and the mean dwell- time asymmetry factor (MDAF) as described in the main manuscript.

Coarse-graining method. We coarse-grain the trajectories of the observed variable (X_1) into 3, 4, 5, 6, and 7 discrete states by spatially dividing the X_1 state space to segments with ratios 1:1:1, $1{:}\frac{1}{2}{:}\frac{1}{2}{:}1$, $1{:}\frac{1}{3}{:}\frac{1}{3}{:}\frac{1}{3}{:}1$, $1{:}\frac{1}{4}{:}\frac{1}{4}{:}\frac{1}{4}{:}\frac{1}{4}{:}1$, $1{:}\frac{1}{5}{:}\frac{1}{5}{:}\frac{1}{5}{:}\frac{1}{5}{:}\frac{1}{5}{:}1$ respectively (as shown in *Figure S2* for *N*=3, 4, 5, 6). The parameters used for the calculations in *Figure S2* are: $F_{max} = 70\ pN$, $T_{eff} = 1.5T$, and $S = 1$. This coarse-graining is used for the results presented in Figure 5 and Figure 6 in the main manuscript.

Mean dwell-time asymmetry factor (MDAF). We calculate the total mean dwell-time asymmetry factor using $\boldsymbol{N^{-1}} \sum \langle \boldsymbol{\tau_{kji}} \rangle / \langle \boldsymbol{\tau_{ijk}} \rangle$, where $\boldsymbol{N}$ is the total number of coarse-grained states. The values of $(\langle \boldsymbol{\tau_{kji}} \rangle / \langle \boldsymbol{\tau_{ijk}} \rangle)$ from transitions among different coarse-grained states are plotted in *Figure S3.*

Tightness of the bounds for different parameters: We calculate the tightness of the bounds for unequal coarse-graining (as shown in *Figure S2*) for 7 coarse-grained states (*N*=7) at different driving parameter values, as shown in Table 1.

Method The steady state averages in Eq. 4 of the form $\langle F \circ \frac{dX}{dt} \rangle = \lim_{t\to\infty} \frac{1}{t} \int_0^t dt' F(t') \circ dx\,(t')$ were calculated using $\langle F \circ \frac{dX}{dt} \rangle \cong \frac{1}{t_{tot}} \sum_{i=1}^{n} \left(\frac{F(t_i)+F(t_{i-1})}{2} \right) \left(X(t_i) - X(t_{i-1}) \right)$, where $t_i = i\Delta t$, and $n = t_{tot}/\Delta t$. We have used $\Delta t = 0.1\ ms$ and the calculation was performed for a trajectory of length $t_{tot} = 100\ s$.

Figure S1

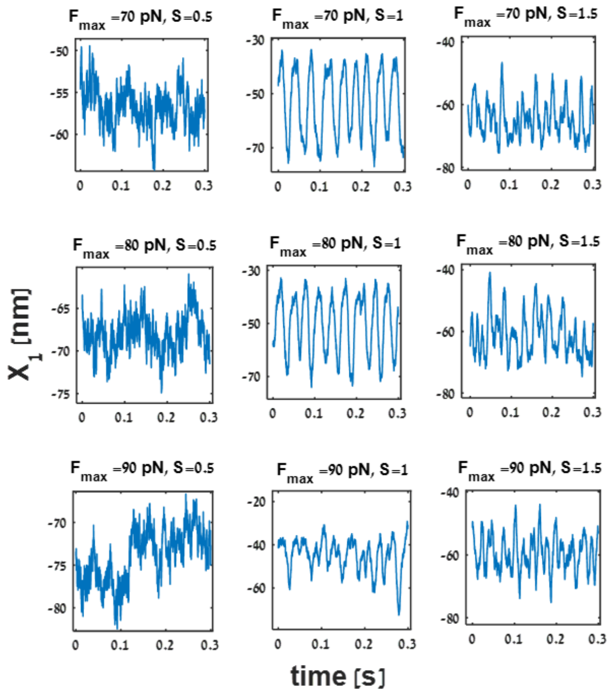

Figure S1. The trajectories of the position of the tip of the hair bundle (X_1) as calculated by solving the coupled differential equations, Eq. 1 and Eq. 2 in the main text for different values of the parameter choices as a function of time. The driving parameter values are written in the subtitles with $T_{eff} = 1.5\,T$. All other parameter values are the same as mentioned in Figure 1 in the main text.

Figure S2.

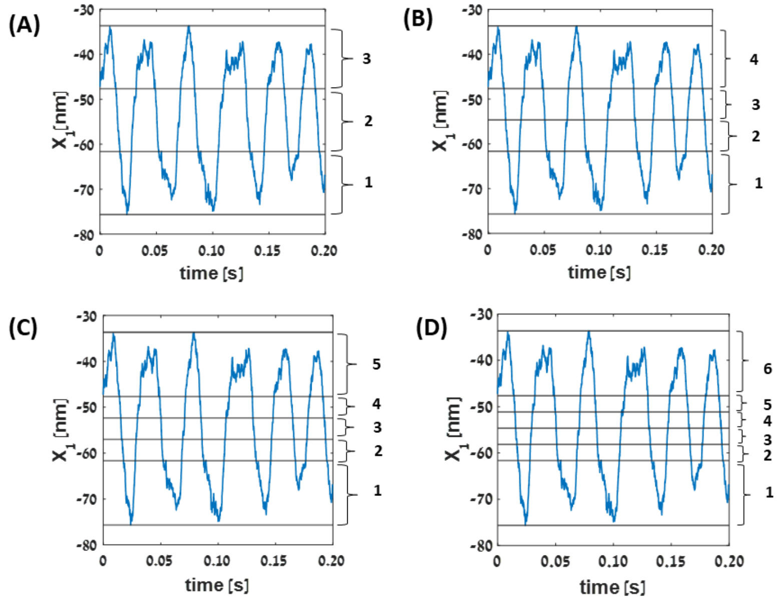

Figure S2. Coarse-graining of the hair bundle tip position ($\boldsymbol{X_1}$) *for parameter values* $\boldsymbol{F_{max} = 70\,pN}$, $\boldsymbol{S = 1}$, $\boldsymbol{T_{eff} = 1.5\,T}$ *. The index numbers on the right side of each panel indicate the number of the coarse-grained states: (A) 3 CG states (1:1:1 division), (B) 4 CG states* ($\mathbf{1}:\frac{1}{2}:\frac{1}{2}:\mathbf{1}$ *division) (C) 5 CG states* ($\mathbf{1}:\frac{1}{3}:\frac{1}{3}:\frac{1}{3}:\mathbf{1}$ *division) and (D) 6 CG states* ($\mathbf{1}:\frac{1}{4}:\frac{1}{4}:\frac{1}{4}:\frac{1}{4}:\mathbf{1}$ *division). Panels (B), (C), and (D) show coarse-graining with unequal division of the* $\boldsymbol{X_1}$ *trajectory as mentioned in the main manuscript. The top and bottom borders are determined by the extremum values of the* $\boldsymbol{X_1}$ *parameter.*

Figure S3.

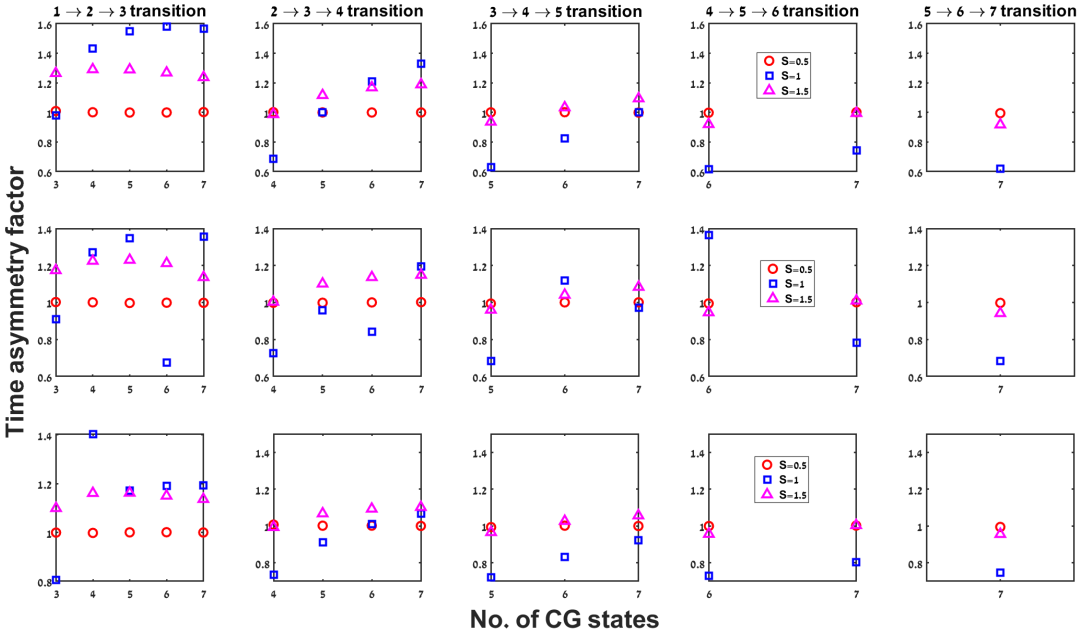

Figure S3. The mean dwell-time asymmetry factors (MDAF, $\langle\tau_{kji}\rangle/\langle\tau_{ijk}\rangle$) as a function of the number of coarse-grained states for different transitions (shown in the subtitles) between the coarse-grained states for different parameter values: $F_{max} = 70\,pN$ (upper panel), $F_{max} = 80\,pN$ (middle panel), $F_{max} = 90\,pN$ (lower panel), $S = 0.5$ (red open circles), $S = 1$ (blue open square), $S = 1.5$ (magenta open triangle)), and $T_{eff} = 1.5\,T$. *All other parameter values are the same as mentioned in Figure 1 in the main text.*

Table 1

The tightness of the bounds for unequal coarse-graining for 7 coarse-grained states at different driving parameter values

Driving parameter values	$\mathbf{EPR_{WTD}/EPR_{tot}}$
$F_{max} = 70\ pN, S = 0.5, T_{eff}/T = 1.5$	0.0666
$F_{max} = 70\ pN, S = 1, T_{eff}/T = 1.5$	0.0024
$F_{max} = 70\ pN, S = 1.5, T_{eff}/T = 1.5$	0.0018
$F_{max} = 80\ pN, S = 0.5, T_{eff}/T = 1.5$	0.1244
$F_{max} = 80\ pN, S = 1, T_{eff}/T = 1.5$	0.0012
$F_{max} = 80\ pN, S = 1.5, T_{eff}/T = 1.5$	0.0024
$F_{max} = 90\ pN, S = 0.5, T_{eff}/T = 1.5$	0.0335
$F_{max} = 90\ pN, S = 1, T_{eff}/T = 1.5$	0.0017
$F_{max} = 90\ pN, S = 1.5, T_{eff}/T = 1.5$	0.0010
